# Development and Elucidation of a Novel Fluorescent Boron-Sensor for the Analysis of Boronic Acid-Containing Compounds

**DOI:** 10.3390/s17102436

**Published:** 2017-10-24

**Authors:** Yoshihide Hattori, Takuya Ogaki, Miki Ishimura, Yoichiro Ohta, Mitsunori Kirihata

**Affiliations:** Research Center of Boron Neutron Capture Therapy, Osaka Prefecture University, 1-1 Gakuen-cho, Nakaku, Sakai, Osaka 599-8531, Japan; takuya.ogaki@riken.jp (T.O.); ishimura@21c.osakafu-u.ac.jp (M.I.); yohta@bioinfo.osakafu-u.ac.jp (Y.O.); kirihata@biochem.osakafu-u.ac.jp (M.K.)

**Keywords:** fluorescent boron sensor, BNCT, boron pharmaceutical, boron(III) complex

## Abstract

Novel boron-containing drugs have recently been suggested as a new class of pharmaceuticals. However, the majority of current boron-detection techniques require expensive facilities and/or tedious pretreatment methods. Thus, to develop a novel and convenient detection method for boron-based pharmaceuticals, imine-type boron-chelating-ligands were previously synthesized for use in a fluorescent sensor for boronic acid containing compounds. However, the fluorescence quantum yield of the imine-type sensor was particularly low, and the sensor was easily decomposed in aqueous media. Thus, in this paper, we report the development of a novel, convenient, and stable fluorescent boron-sensor based on *O*- and *N*-chelation (i.e., 2-(pyridine-2yl)phenol), and a corresponding method for the quantitative and qualitative detection of boronic acid-containing compounds using this commercially available sensor is presented.

## 1. Introduction

**Figure 1:**
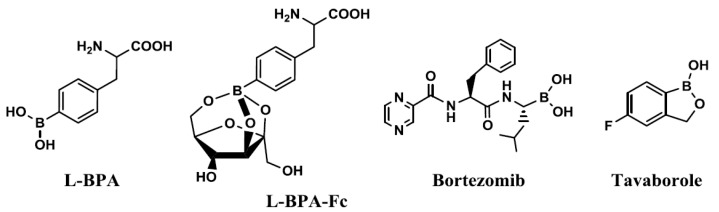
A selection of boron-containing pharmaceuticals.

The use of boronic acid-containing compounds has recently received growing interest in a range of fields, such as materials science, analytical chemistry, chemical biology, and pharmacology. In particular, a large number of studies focusing on the interactions between boron compounds and biomolecules such as sugars, proteins and peptides have been reported in recent years [1,2,3,4,5,6]. For example, in the field of pharmacology, boronic acid-containing compounds have been developed as boron carriers for boron neutron capture therapy (BNCT) [7], and as antibacterial agents, protease inhibitors, sugar sensors, and cell-penetrating peptides. In addition, new boron-containing drugs, including *p*-borono-L-phenylalanine (L-BPA, for BNCT), L-BPA-fructose complex (L-BPA-Fc) [8,9,10], bortezomib (for the treatment of multiple myeloma) [11,12], and tavaborole (an antifungal drug for the treatment of onychomycosis) [13] have been suggested as a new class of pharmaceuticals [14,15] (Figure 1).

During the development of novel pharmaceutical compounds, drug distribution analysis is of particular importance. For example, the distribution of boron-based pharmaceuticals in tumor tissue and normal tissue must be determined to elucidate the suitability of pharmaceuticals for BNCT, and monitoring of the boron concentration in bloodstream is of key importance in the BNCT treatment process. In this context, evaluation of the concentration of boron-based pharmaceuticals in biological environments should be possible through the detection of boron, since biological tissues generally contain little or no boron. However, the majority of boron detection methods, such as those based on prompt gamma-ray analysis (PGA) and inductively coupled plasma optical emission spectrometry (ICP-OES) are costly, and often require extensive sample pretreatment.

**Figure 2:**
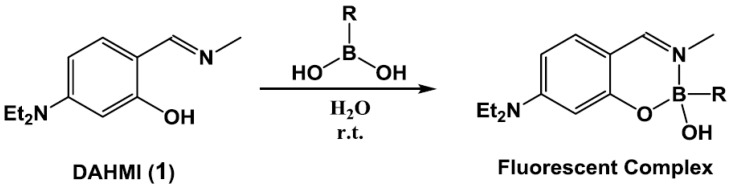
Complex formation using the previously reported fluorescent boron sensor DAHMI (**1**).

In this context, we recently designed and synthesized a novel fluorescent probe for boronic acid, namely DAHMI (**1**) (Figure 2) [16]. This probe reacted rapidly with boron-based pharmaceuticals in aqueous media, resulting in the emission of blue fluorescence by the formed DAHMI-boron complexes. Furthermore, DAHMI allowed visualization of the distribution of boron-containing pharmaceuticals such as L-BPA, and tavabolore in live tumor cells without complicated pretreatment [17]. However, the fluorescence quantum yields of the resulting DAHMI-boron complexes were extremely low (i.e., <1%), and DAHMI was easily decomposed by hydrolysis in aqueous media. As such, DAHMI is not particularly suitable for use as a boron sensor in the quantitative analysis of boron compounds in biological samples.

Thus, in this paper, we report the development of a novel, convenient, and stable fluorescent probe for boronic acid-containing compounds, and present a subsequent elucidation of the fluorescence properties of the resulting sensor-boronic acid complexes.

## 2. Results and Discussion

In order for a fluorescent sensor to detect boronic acid derivatives in biological environments, the following criteria must be met: (i) rapid reaction with boronic acid at room temperature; (ii) a change in emission upon complexation with boronic acid; (iii) fluorescence in water; and (iv) stability in water.

**Figure 3:**
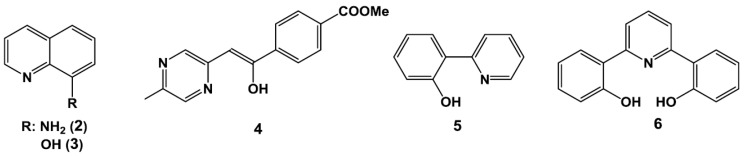
Candidate compounds for the fluorescent boron sensor.

**Figure 4:**
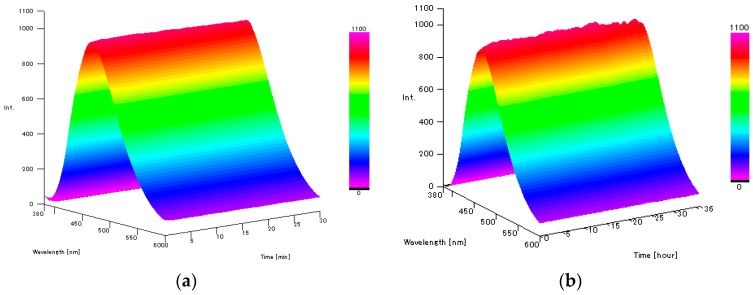
Variation in the fluorescence spectra of the 5-BPA complex in 50 vol % DMSO/PBS (0.2 mM, excitation wavelength: 355 nm) at 25 °C. (**a**) prepared after 0–60 min; (**b**) prepared after 0–36 h.

Thus, we herein employed a range of commercially available and easily prepared chelating ligands **2**–**6** as precursors for the boron sensor (Figure 3) [18,19,20,21]. These ligands were considered suitable as they were both soluble and stable in a 50 vol % DMSO/H_2_O solution, and because they emit very weak fluorescence in aqueous solutions. To confirm the potential of our previously prepared fluorescent probe DAHMI in addition to the five chelating ligands for use as boron sensors, we measured the fluorescence spectra of compounds **1**–**6** and their corresponding BPA complexes in a 50 vol % DMSO/PBS (phosphate-buffered saline) solution at 25 °C (Table 1). Although compounds **2** and **6** did not react with BPA under these conditions, DAHMI and compounds **3**–**5** reacted to give fluorescent complexes with BPA. This result suggests that *N*, *O*-type chelating ligands are suitable for use as fluorescent boron-sensors. In addition, we observed that the excitation and fluorescence wavelengths of these complexes differed from those of the corresponding free ligands. In particular, the fluorescence wavelength of the **5**-BPA (**5** = 2-(pyridine-2-yl)phenol) complex was blue shifted by ~20 nm, and the Stokes shift of the complex was extremely large. Furthermore, the maximum fluorescence quantum yield was observed for **5**-BPA, with a 15-fold higher yield being observed than for the DAHMI-BPA complex. Indeed, this **5**-BPA complex showed particular potential for use as a sensor, as it was prepared rapidly by the simple mixing of a solution of **5** with a solution of BPA (1.0 eq.) at room temperature in 50 vol % DMSO/PBS. Upon mixing, the fluorescence intensity of **5**-BPA complex was reached a maximum within a few seconds, and this complex was stable in solution over 24 h (Figure 4). These results indicate that compound **5** is suitable for use as a fluorescent sensor in the detection of boronic acid-containing compounds.

**Figure 5:**
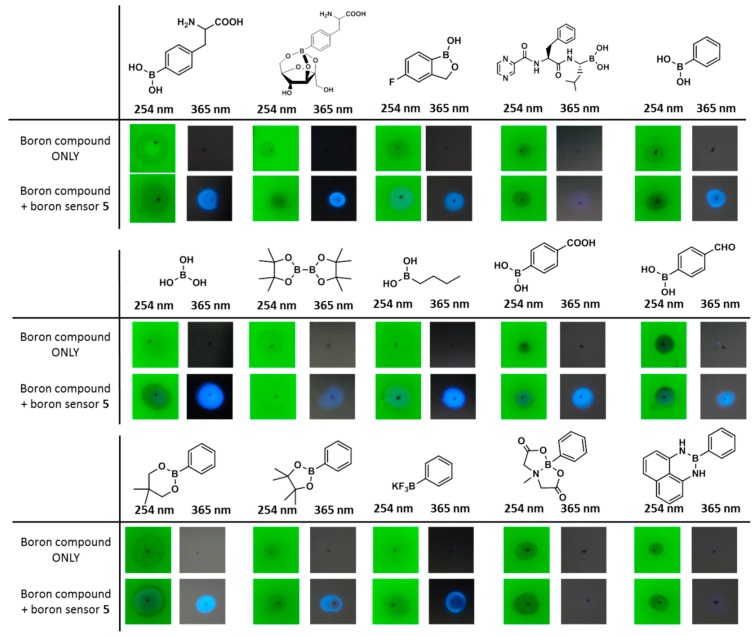
Staining test of various boron-containing compounds spotted onto silica-gel plates followed by the addition of boron sensor **5** (viewed by illumination at 254 or 365 nm using a handheld UV lamp).

**Figure 6:**
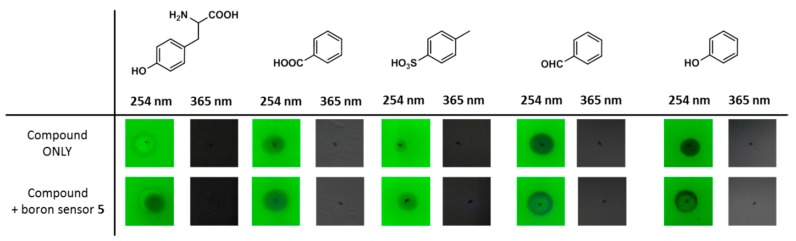
Staining test of boron-free compounds spotted onto silica-gel plates followed by the addition of boron sensor **5** (viewed by illumination at 254 or 365 nm using a handheld UV lamp).

Thus, to confirm the potential of boron sensor **5** for use in the qualitative analysis of boronic acid-containing compounds, a number of boron compounds were spotted onto silica gel plates and stained using a 1.0 mM solution of **5** in acetone (Figure 5). Although boron sensor **5** was visible under irradiation using a 254 nm hand-held UV lamp, the spot of **5** did not emit fluorescence under 254 or 365 nm UV lamps. Thus, as shown in Figure 5, all spots corresponding to the different boronic acid-containing compounds, including L-BPA, bortezomib and tavaborole produced bright blue fluorescence using a standard short-wave UV lamp (365 nm) following staining with compound **5**. In particular, boronic ester-type protected derivatives, such as pinacolato, 1,3-dihydroxydimetyl, trifluoroborate and L-BPA-Fc, exhibited strong fluorescence upon complexation with **5**. However, in the case of boronic amide type protected derivatives, such as *N*-methyliminodiacetic acid and 1,8-diaminonaphtnalene, only weak fluorescence was observed. As a control, a number of boron-free compounds were also spotted onto the silica plates, but no fluorescence was observed under UV irradiation at either 254 or 365 nm (Figure 6). These results therefore suggest that boron sensor **5** is a useful tool for the qualitative analysis of boronic acid-containing compounds.

**Figure 7:**
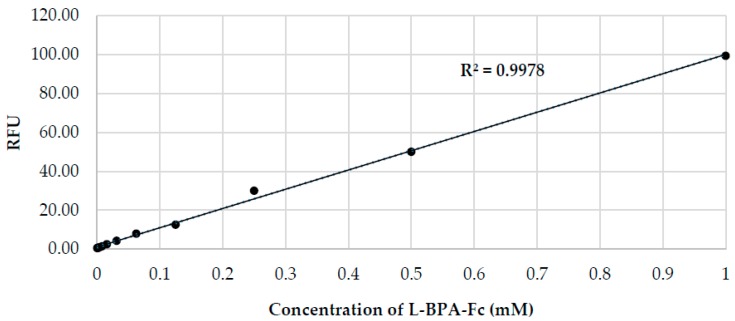
Effect of L-BPA-Fc concentration on the fluorescence intensity following staining with boron-sensor **5** (10 mM in 50 vol % DMSO/PBS, ex λ: 360 nm, em λ: 460 nm).

Finally, to determine the potential of boron sensor **5** in the quantitative analysis of boron pharmaceuticals, we examined the relationship between the concentration of L-BPA-Fc and the emission of the complex upon staining with **5** in 50 vol % DMSO/PBS using a standard plate reader. As shown in Figure 7, the emission intensity correlated positively with the concentration of L-BPA-Fc, thereby indicating that boron sensor **5** is suitable for use in the quantitative analysis of L-BPA-Fc (0.5–1000 μM, 0.005–10 Bppm, R^2^ > 0.99). Furthermore, the detection limit of this method was comparable to ICP-OES.

## 3. Conclusions

We herein developed and elucidated a novel efficient and commercially available fluorescent sensor based on *O*- and *N*- chelation (i.e., 2-(pyridine-2yl)phenol) for the analysis of boronic acid-containing compounds. We found that this boron sensor reacts rapidly with boronic acid at room temperature, and that it selectively detects boronic acid-containing compounds. Furthermore, the quantitative analyses of boronic acid-containing compounds using this sensor were carried out using a standard plate reader, with the compounds of interest being detected in concentrations of 0.5–1000 μM. These results therefore suggest that this fluorescent boron sensor is suitable for use in the qualitative and quantitative analysis of boronic acid-containing compounds. We therefore expect that the system reported herein will be applicable in the detection of boron-based pharmaceuticals, with the potential to replace current expensive and tedious analytical methods.

## 4. Experimental Section

### 4.1. Genaral

L-BPA was provided by the Stella Pharma Corporation (Osaka, Japan), while bortezomib and tavaborole were purchased from Cosmo Bio Co., Ltd. (Tokyo, Japan). Compounds **2**, **3**, and **5** were purchased from Wako Pure Chemical Industries, Ltd. (Osaka, Japan). Compounds **4** and **6** were prepared according to a previous literature method [19,21]. Fluorescence spectra were measured on a FP-8200 spectrometer (JASCO Corporation, Tokyo, Japan). Absolute quantum yields were determined by the Hamamatsu C9920-01 calibrated integrating sphere system (Hamamatsu Photonics K.K., Shizuoka, Japan).

### 4.2. Preparation of the Sensor-BPA Complexes

A solution of boron sensor (1.0 mM in DMSO, 2.5 mL) was added to a solution of BPA (1.0 mM in PBS buffer, 2.5 mL). After allowing this mixture to stand for 10 min at 37 °C, the resulting solution was employed for fluorescence measurement.

### 4.3. Silica Gel Plate Staining Using Boron Sensor 5

L-BPA and L-BPA-Fc were prepared as aqueous solutions (10 mM) concentrations while solutions of all other boron-containing compounds were prepared in acetone (10 mM). Each sample solution (5 μL) was applied to a glass-backed silica-gel TLC plate containing the F254 fluorescent indicator (Merck KGaA, Darmstadt, Germany) and allowed to dry under air at room temperature prior to treatment of the test compound spot with a solution of boron sensor 5 (1.0 mM in acetone). After allowing to dry once again under air at room temperature, the plates were visualized by illumination at either 254 or 365 nm using a handheld UV lamp, and photographic images were recorded.

### 4.4. Effect of BPA Concentration on the Emitted Fluorescence Following Staining with 5

To a 96-well microplate (FluoroNuncTM flat bottom black polystyrene plate, Thermo Fisher Scientific, Waltham, MA) were added of a L-BPA-Fc solution of the desired concentration (50 μL, 0.5–1000 μM in H_2_O) and a solution of boron sensor 5 (50 μL, 10 mM in DMSO. After mixing for 5 min at 37 °C, the fluorescence intensity was measured using a Fluoroskan Ascent FL microplate reader (Thermo Fisher Scientific, Waltham, MA, USA).

## Figures and Tables

**Table 1:** Fluorescence properties of the various ligands and ligand-BPA complexes ^[a]^.

Ligand	Ligand Only				Ligand-BPA Complex			
	Ex Max (nm)	Em Max (nm)	Stokes Shift (cm^−1^)	*φ*	Ex Max (nm)	Em Max (nm)	Stokes Shift (cm^−1^)	*φ*
**1**	413	551	6064	—	408	430	1254	0.6%
**2**	374	531	—	—	374	531	—	—
**3**	419	477	2902	—	421	516	4373	3.3%
**4**	340	384	3370	—	352	433	5314	1.2%
**5**	376	483	5892	—	355	464	6617	9.3%
**6**	376	—	—	—	376	—	—	—

^[a]^ Measured at a concentration of 1.0 mM in 50 vol % DMSO/PBS at 25 °C.

## Supplementary Materials

The following are available online at http://www.mdpi.com/1424-8220/17/10/2436/s1.
