# Supplementary material for: Development and Elucidation of a Novel Fluorescent Boron-Sensor for the Analysis of Boronic Acid-Containing Compounds
Source: Sensors (Basel). 2017 Oct 24;17(10):2436. doi: 10.3390/s17102436 (PMC5677422; doi:10.3390/s17102436)

Supporting Information

Development and Elucidation of a Novel Fluorescent Boron-Sensor for the Analysis of Boronic Acid containing Compounds

Yoshihide Hattori,[1,] Takuya Ohgaki,[1] Miki Ishimura,[1] Yoichiro Ohta,[1] Hiroshi Takenaka,[1] and Mitsunori Kirihata[1]

[1]Research Center of Boron Neutron Capture Therapy, Osaka Prefecture University, 1-1 Gakuen-cho, Nakaku, Sakai, Japan.

Table of contents

1. NMR Spectrum

1-1. General

1-2. Preparation of boron-sensor-PhB(OH)$_2$ complex

1-3. NMR Assignment

1-4. NMR Spectrum

Figure S1a-c: NMR spectrum of phenyl boric acid

Figure S2a-c: NMR spectrum of compound 3

Figure S3a-c: NMR spectrum of 3-PhB(OH)$_2$ complex

Figure S4a-c: NMR spectrum of compound 4

Figure S5a-c: NMR spectrum of compound 4-PhB(OH)$_2$ complex

Figure S6a-c: NMR spectrum of compound 5

Figure S7a-c: NMR spectrum of compound 5-PhB(OH)$_2$ complex

1-1. General

^{1}H NMR spectra were measured on a JNM-ECZR (500 MHz, JEOL Ltd., Tokyo, Japan). The chemical shifts in ^{1}H NMR are given in δ values from TMS.

1-2. Preparation of boron-sensor-PhB(OH)2 complex

To a solution of PhB(OH)$_2$ (0.15 mmol) in acetone-d6 (0.25 mL) was added a solution of boron-sensor (0.15 mmol) in acetone-d6 (0.25 mL). The mixture was permitted to stand for 20 min at 25 °C, the solution was used for measurement of NMR (25 °C).

1-3. NMR assignment

Phenyl boric acid

^{1}H-NMR (500 MHz, ACETONE-D6) δ 7.88 (dd, J = 8.0, 1.1 Hz, 2H), 7.40-7.43 (m, 1H), 7.33-7.36 (m, 2H), 7.16 (s, 2H).

^{13}C-NMR (126 MHz, ACETONE-D6) δ 134.89, 131.03, 128.24

8-Quinolinol (3)

^{1}H-NMR (500 MHz, ACETONE-D6) δ 8.84 (q, J = 1.9 Hz, 1H), 8.33 (dd, J = 8.3, 1.4 Hz, 1H), 7.56 (q, J = 4.0 Hz, 1H), 7.49 (t, J = 8.0 Hz, 1H), 7.43 (dd, J = 8.6, 1.1 Hz, 1H), 7.15 (dd, J = 7.4, 1.1 Hz, 1H).

^{13}C-NMR (126 MHz, ACETONE-D6) δ 153.64, 149.02, 139.23, 136.97, 129.63, 128.49, 122.88, 118.65, 110.84

3-PhB(OH)$_2$ complex

^{1}H-NMR (500 MHz, ACETONE-D6) δ 8.83 (t, J = 2.0 Hz, 1H), 8.31-8.33 (m, 1H), 7.90 (d, J = 6.9 Hz, 2H), 7.55 (q, J = 4.2 Hz, 1H), 7.49 (t, J = 8.0 Hz, 1H), 7.40-7.43 (m, 2H), 7.35 (t, J = 7.2 Hz, 2H), 7.14-7.26 (m, 2H)

^{13}C-NMR (126 MHz, ACETONE-D6) δ153.63, 148.98, 141.43, 139.21, 136.96, 134.90, 132.21, 132.02, 131.01, 129.61, 128.47, 128.23, 122.85, 118.64, 110.88

Methyl (Z)-4-(1-hydroxy-2-(5-methylpyrazin-2-yl)vinyl)benzoate (4)

^{1}H-NMR (500 MHz, ACETONE-D6) δ 8.62-8.30 (2H), 8.29-7.92 (5H), 6.73-6.42 (1H), 4.92-4.84 (0H), 4.72-4.63 (0H), 4.07-3.79 (4H), 2.97-2.73 (2H), 2.71-2.38 (3H)

^{13}C-NMR (126 MHz, ACETONE-D6) δ166.74, 166.45, 150.69, 145.57, 144.43, 144.36, 142.29, 141.14, 140.02, 131.66, 130.52, 130.42, 130.30, 129.37, 126.16, 126.05, 94.88, 94.77, 52.70, 52.41, 45.72, 21.34, 21.08, 20.94

2-(pyridine-2yl)phenol (5)

^{1}H-NMR (500 MHz, METHANOL-D4) δ 8.52 (d, J = 4.0 Hz, 1H), 8.05 (d, J = 8.0 Hz, 1H), 7.90 (qd, J = 7.9, 1.4 Hz, 2H), 7.26-7.33 (m, 2H), 6.89-6.93 (m, 2H)

^{13}C-NMR (126 MHz, METHANOL-D4) δ 180.15, 177.82, 166.12, 158.45, 151.38, 146.57, 146.52, 142.05, 139.41, 138.69, 138.22

5- PhB(OH)$_2$ complex

^{1}H-NMR (500 MHz, METHANOL-D4) δ 8.58 (d, J = 5.7 Hz, 1H), 8.18-8.25 (m, 2H), 7.88-7.90 (m, 1H), 7.59-7.62 (m, 1H), 7.42-7.45 (m, 1H), 7.29 (d, J = 6.3 Hz, 2H), 7.08-7.15 (m, 4H), 6.95 (t, J = 7.3 Hz, 1H)

^{13}C-NMR (126 MHz, METHANOL-D4) δ 159.11, 151.82, 143.66, 143.38, 135.25, 133.09, 128.50, 128.31, 127.98, 127.01, 124.44, 122.15, 121.30, 120.94, 118.91

1-4. NMR Spectrum

Figure S1a. ^{1}H NMR of phenylboric acid (acetone-d6)

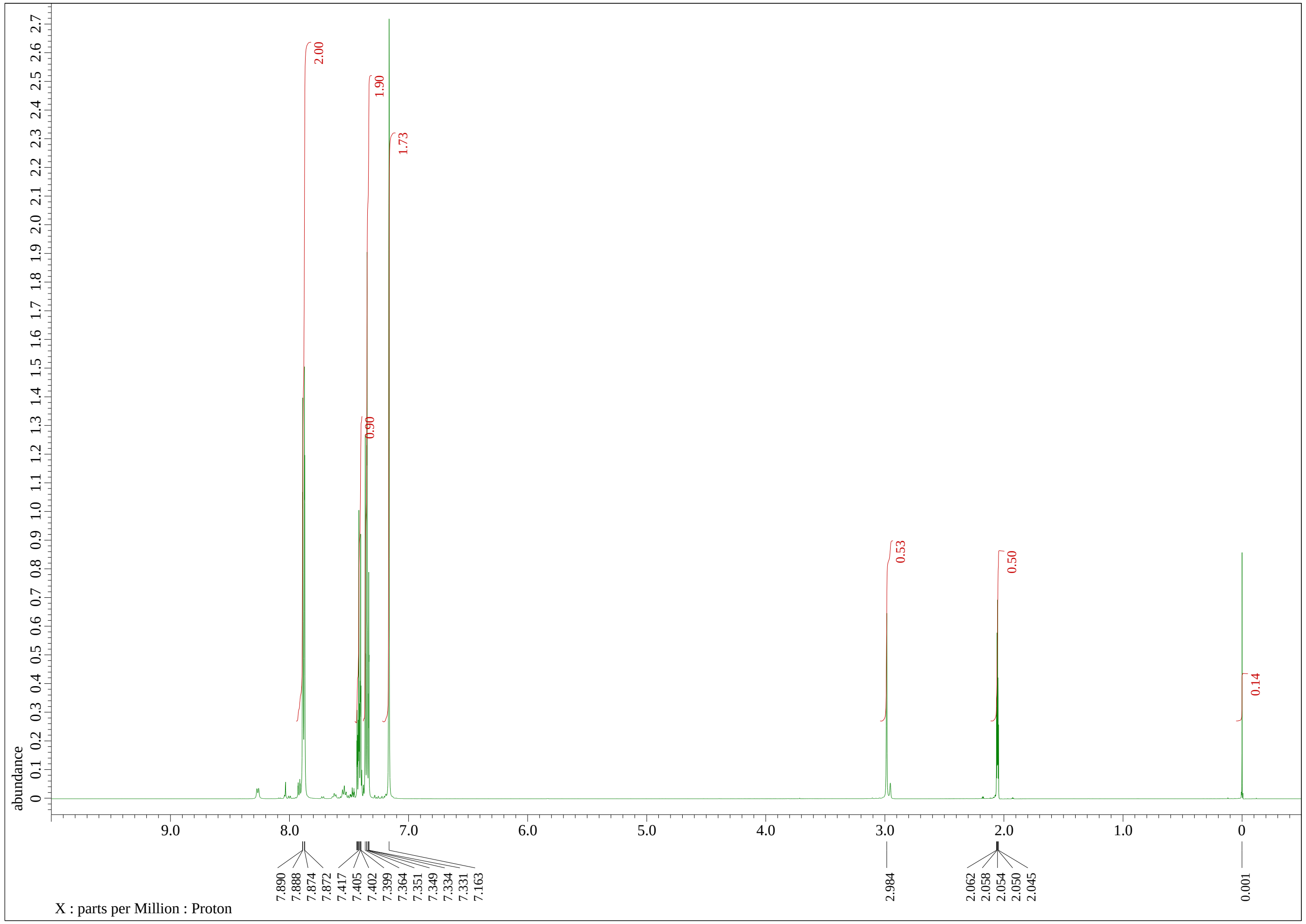

Figure S1b. ^{1}H NMR of phenylboric acid (acetone-d6, expansion (6.0-9.0ppm))

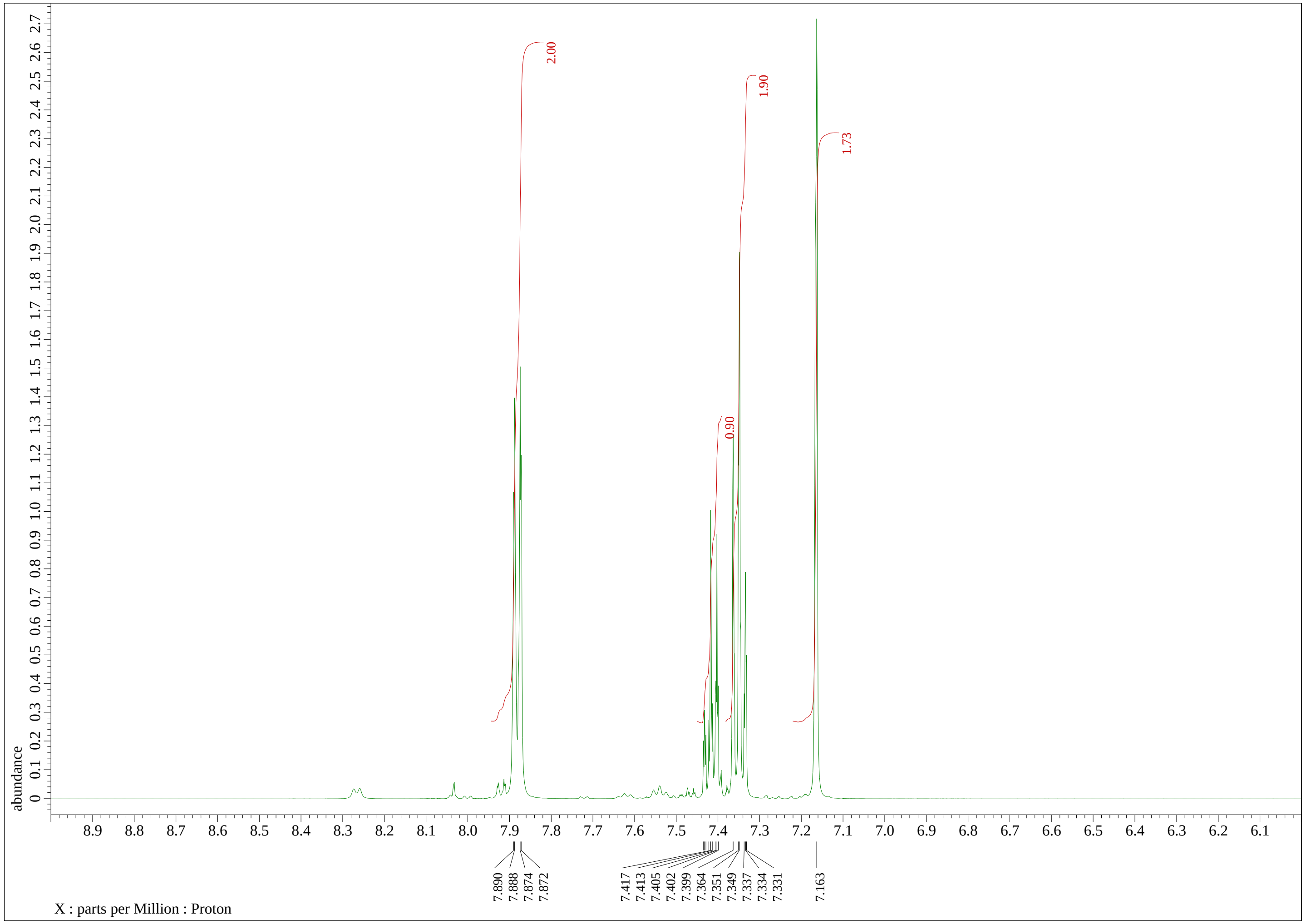

Figure S1c. ^{13}C NMR of phenylboric acid (acetone-d6)

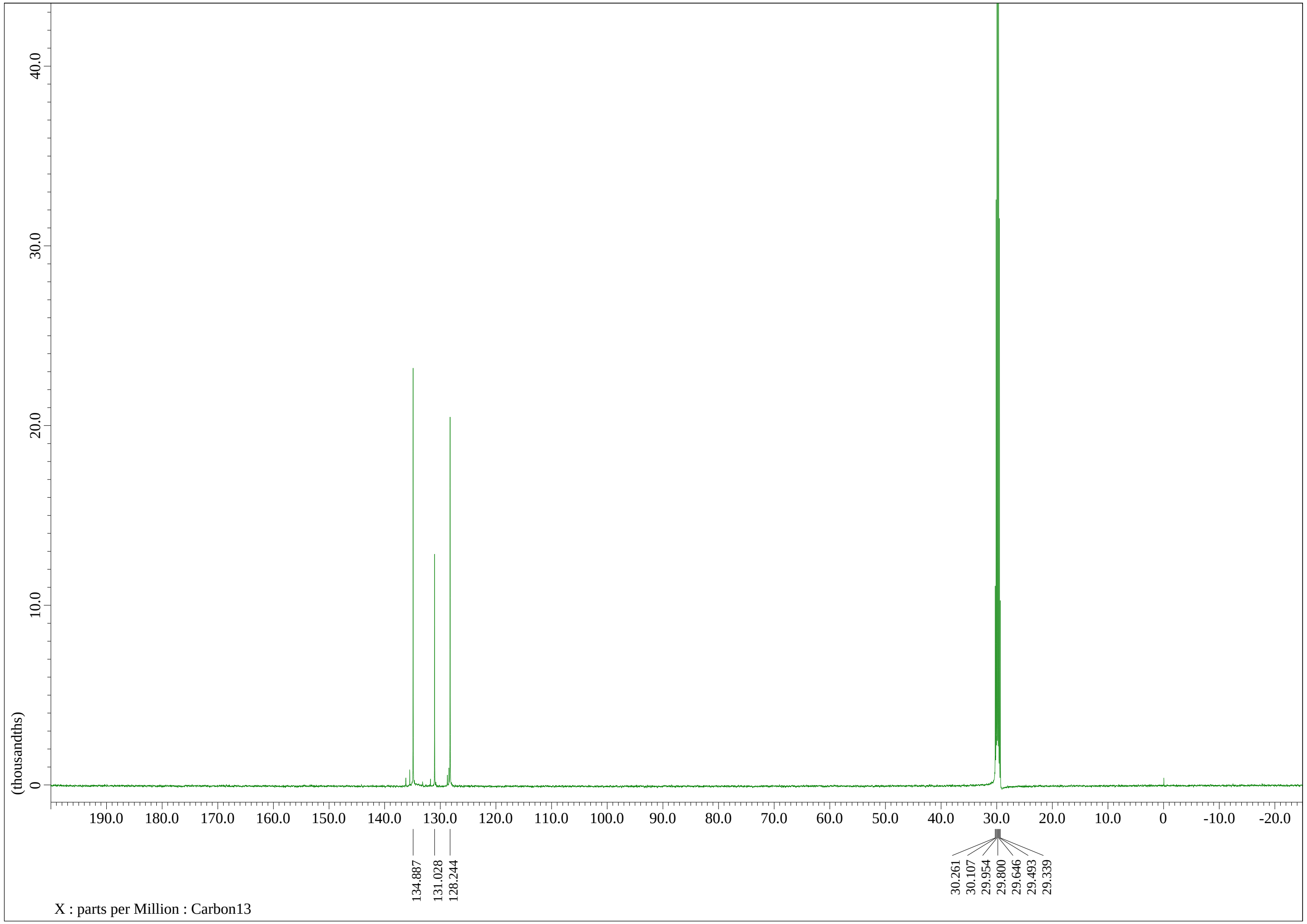

Figure S2a. ^{1}H NMR of 3 (acetone-d6)

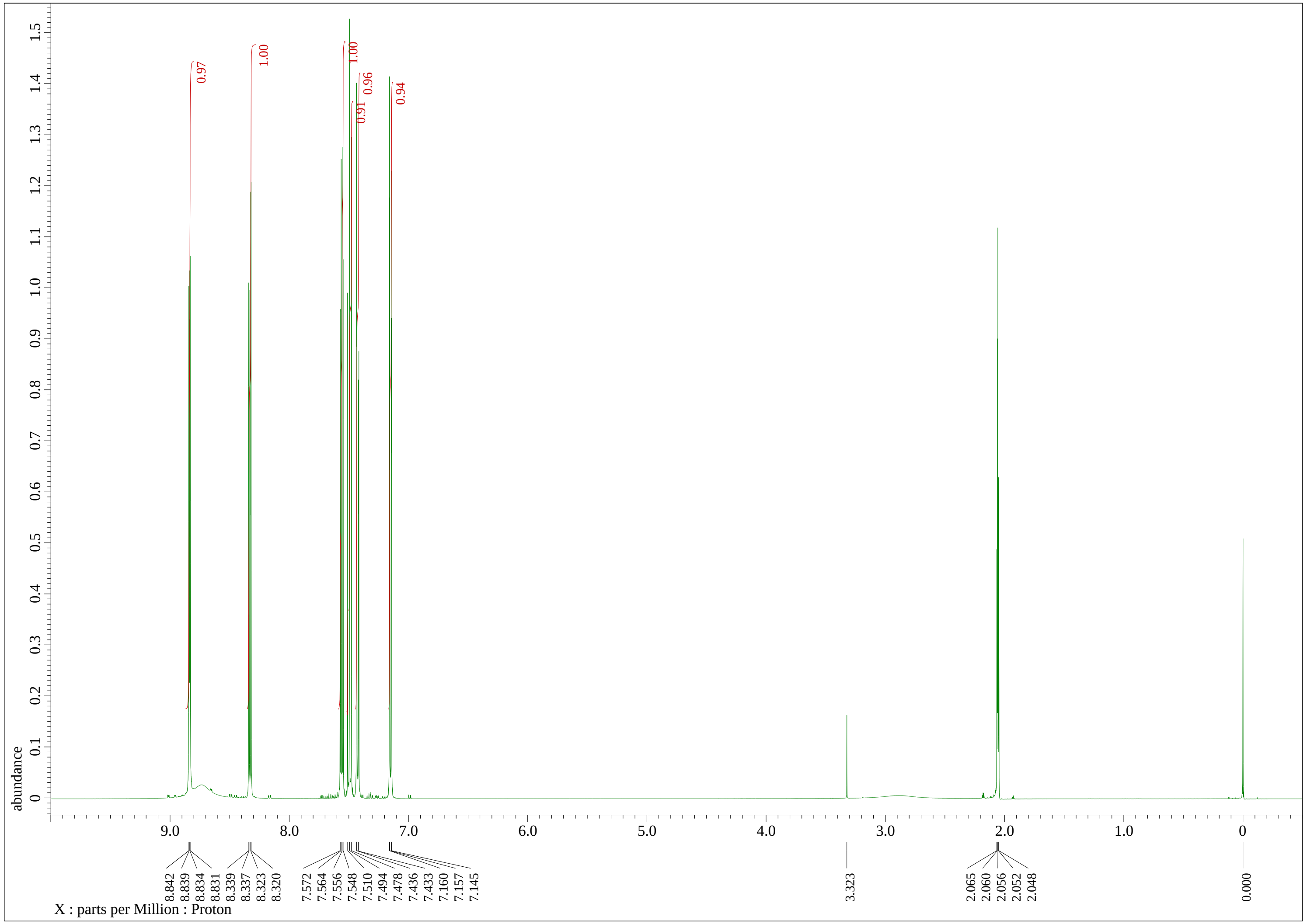

Figure S2b. ^{1}H NMR of 3 (acetone-d6, expansion (6.0-9.0ppm))

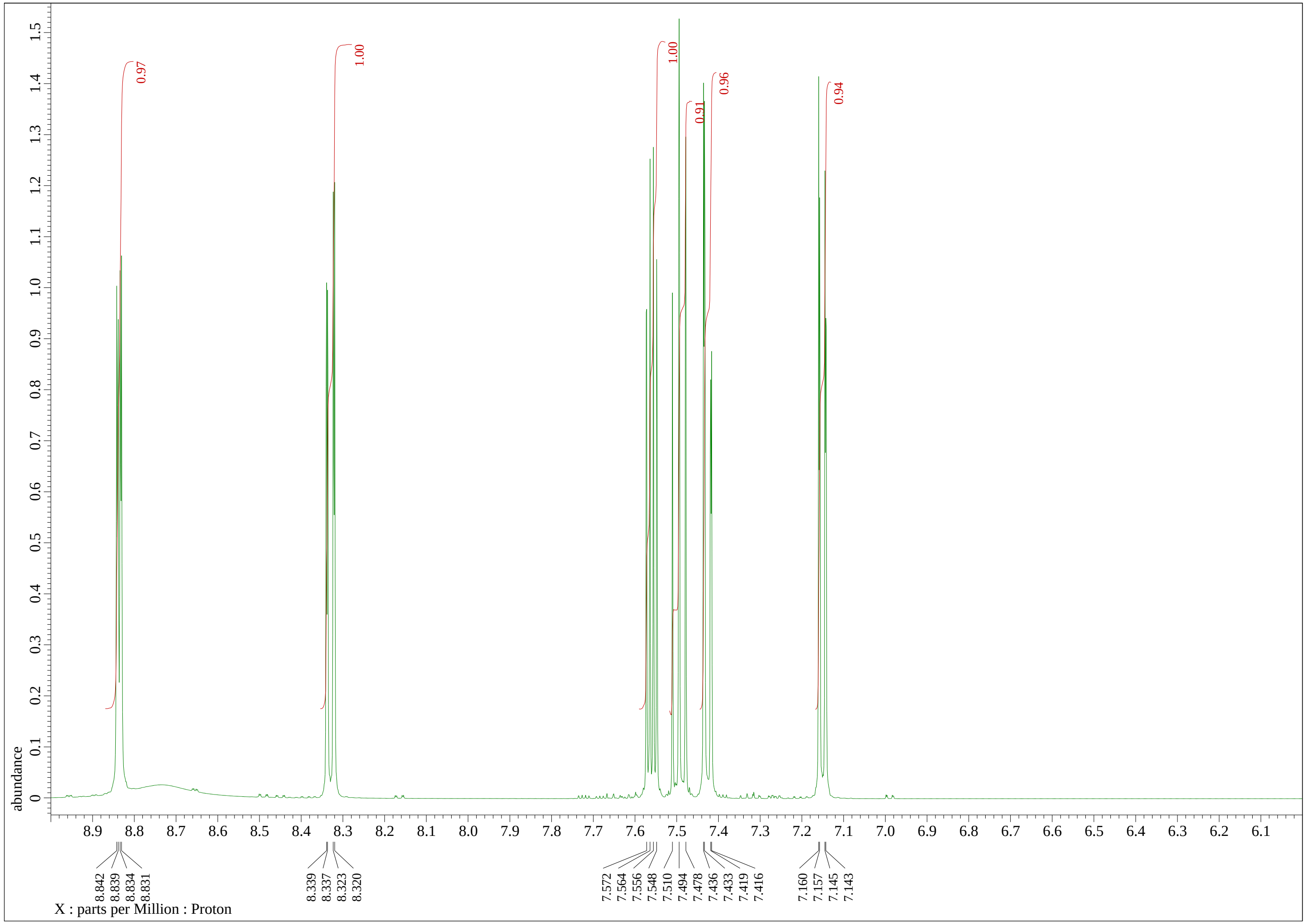

Figure S2c. ^{13}C NMR of 3 (acetone-d6)

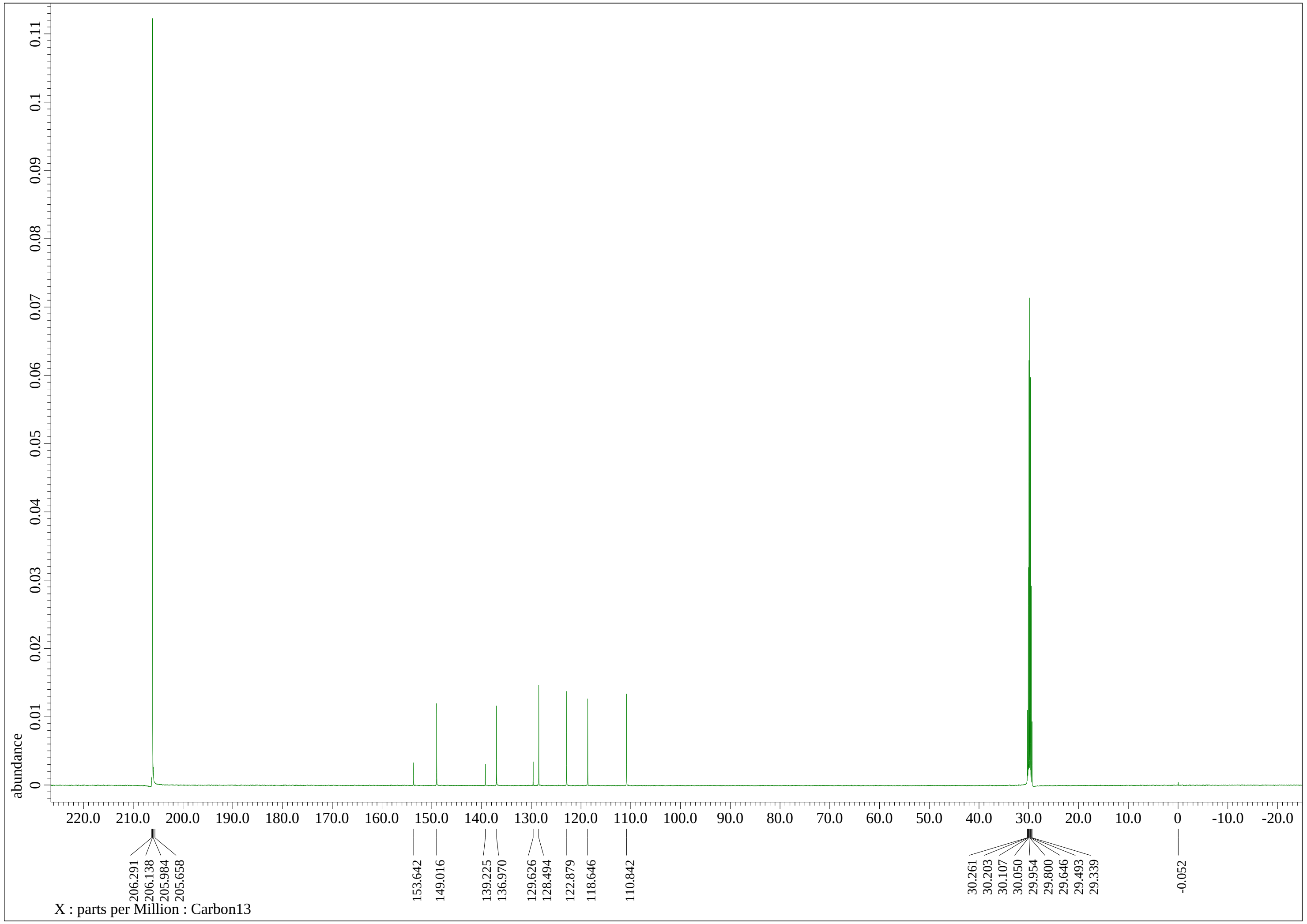

Figure S3a. ^{1}H NMR of 3-PhB(OH)$_2$ complex (acetone-d6)

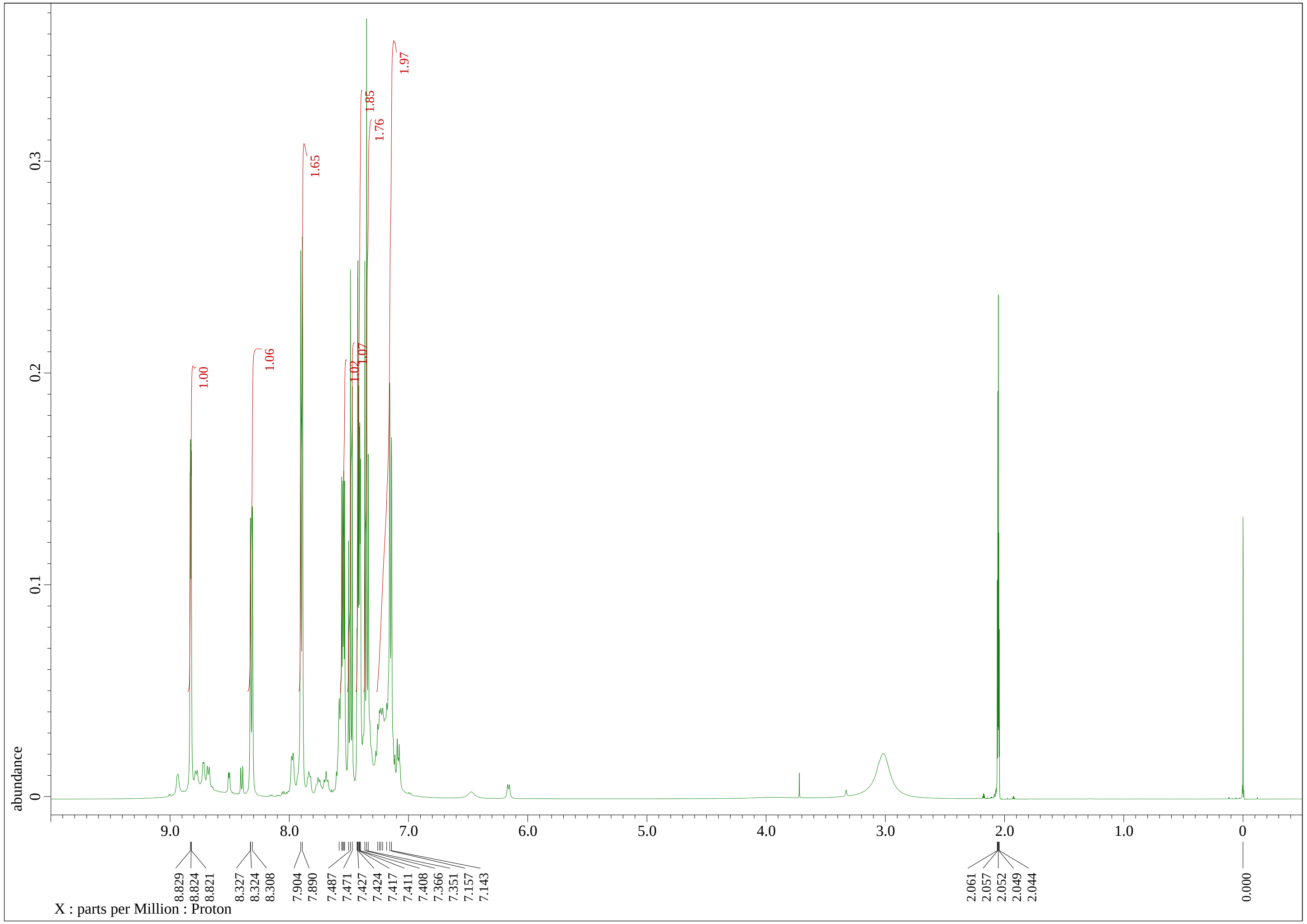

Figure S3b. ^{1}H NMR of 3-PhB(OH)$_2$ complex (acetone-d6, expansion 6.0-9.5ppm)

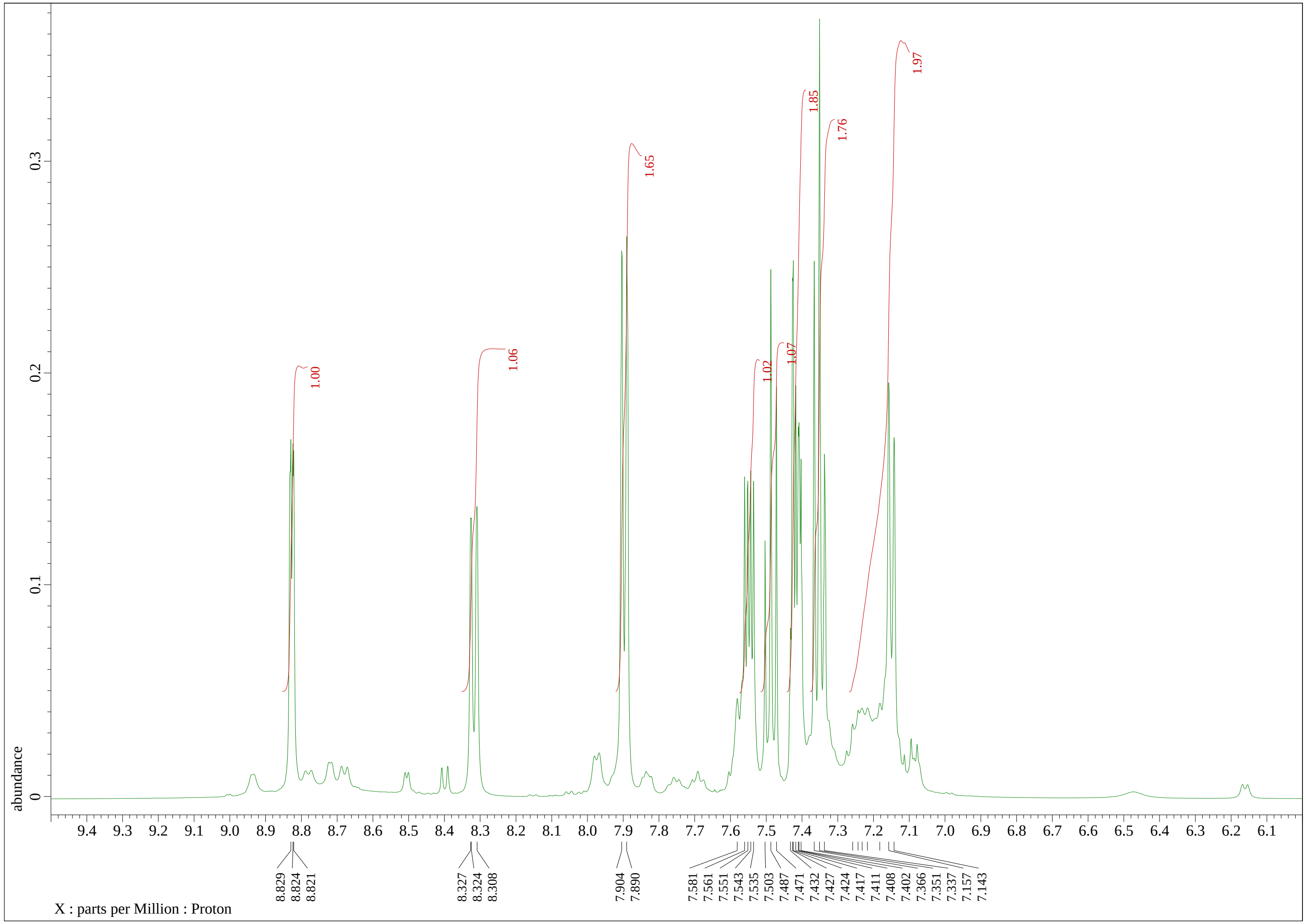

Figure S3c. ^{13}C NMR of 3-PhB(OH)$_2$ complex (acetone-d6)

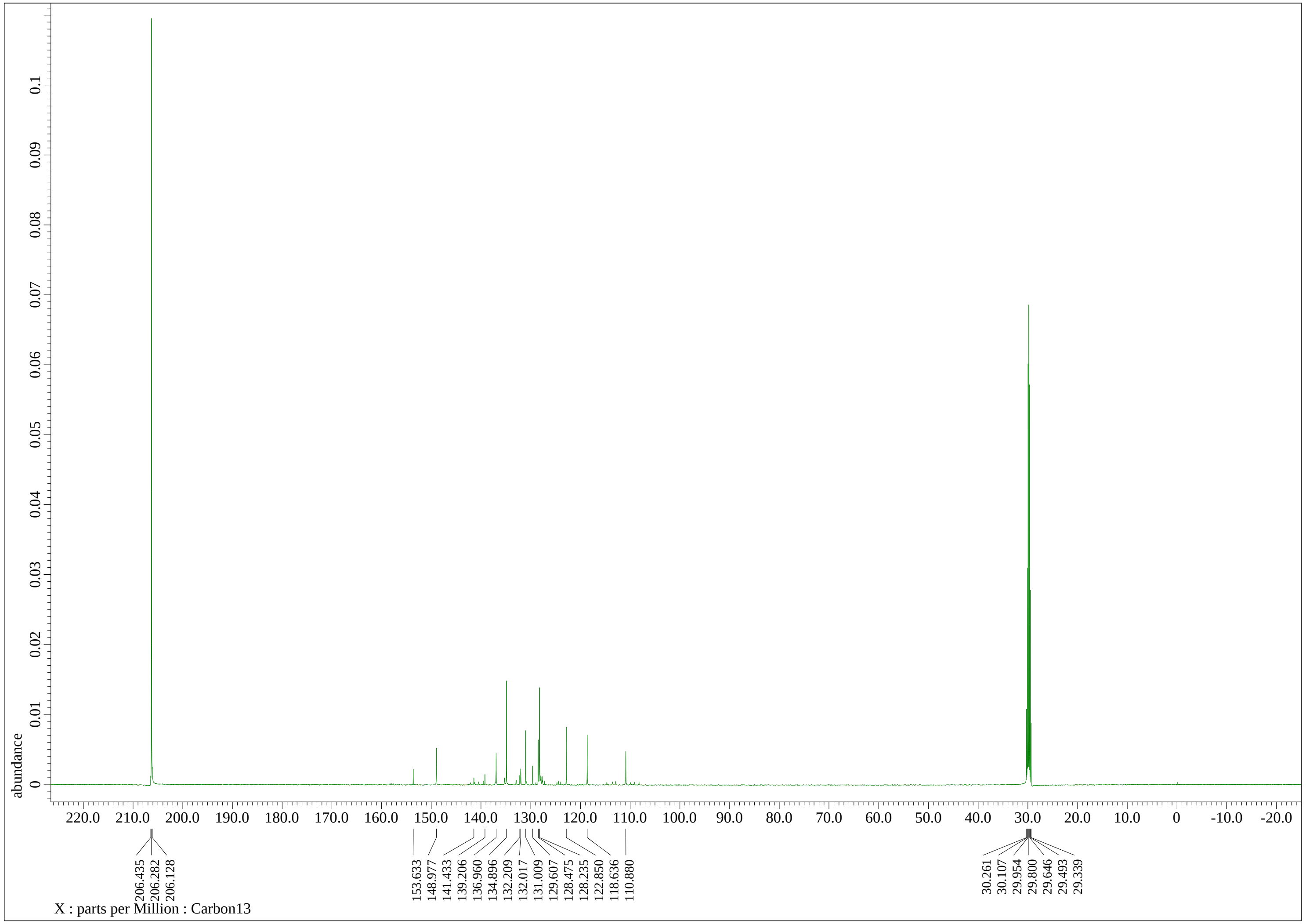

Figure S4a. ^{1}H NMR of 4 (acetone-d6)

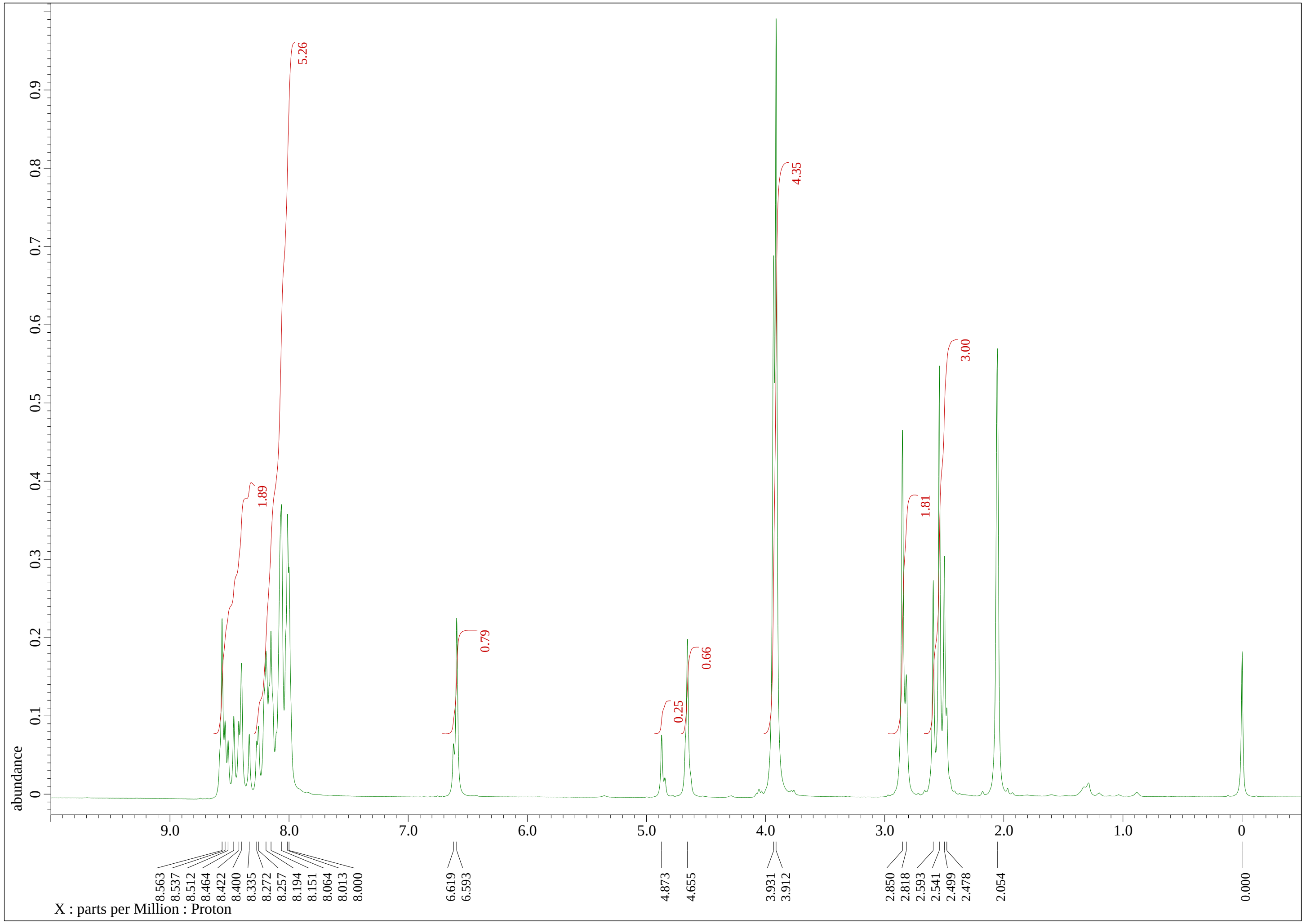

Figure S4b. ^{1}H NMR of 4 (acetone-d6, expansion (6.0-9.0ppm))

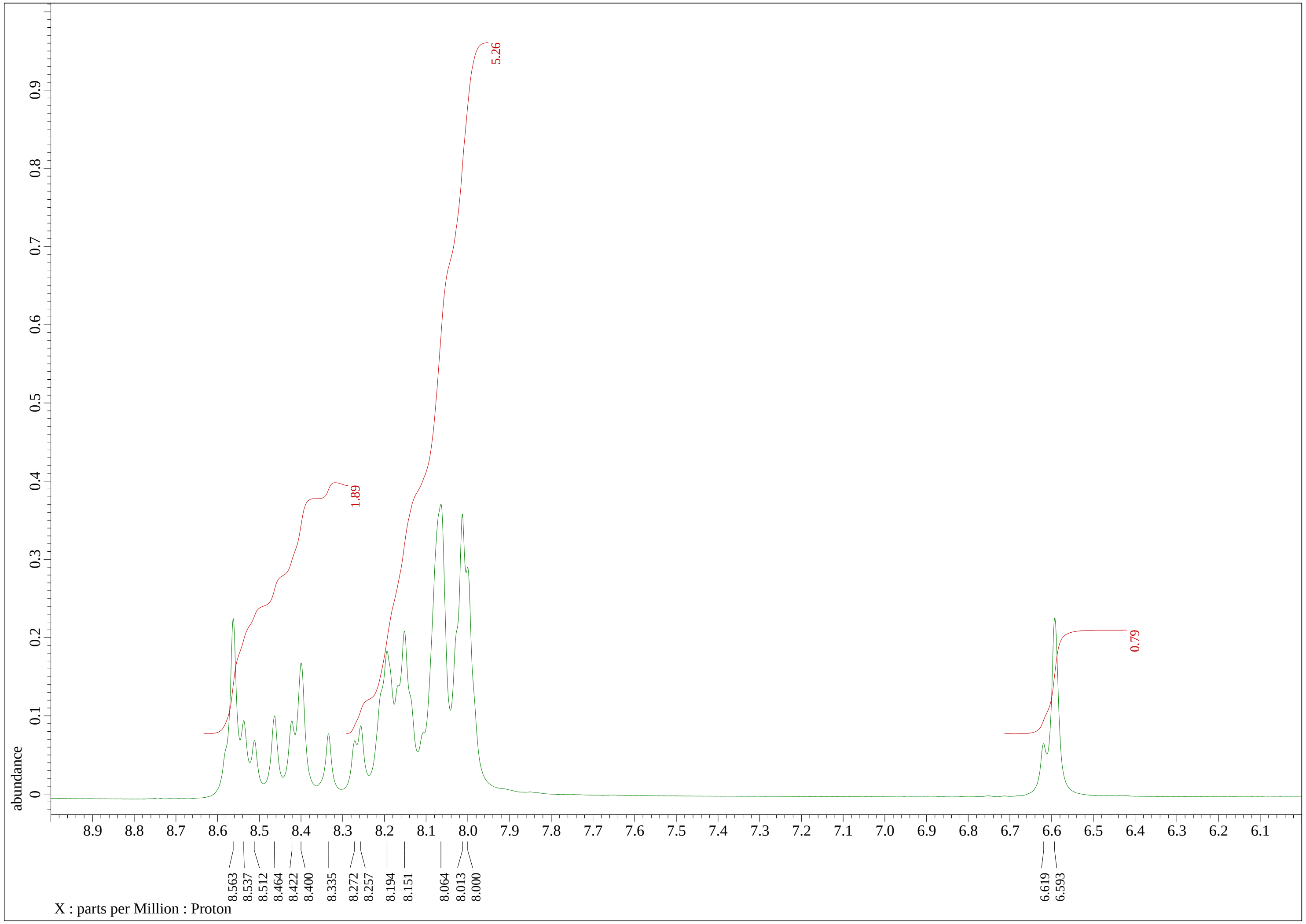

Figure S4c. ^{13}C NMR of 4 (acetone-d6)

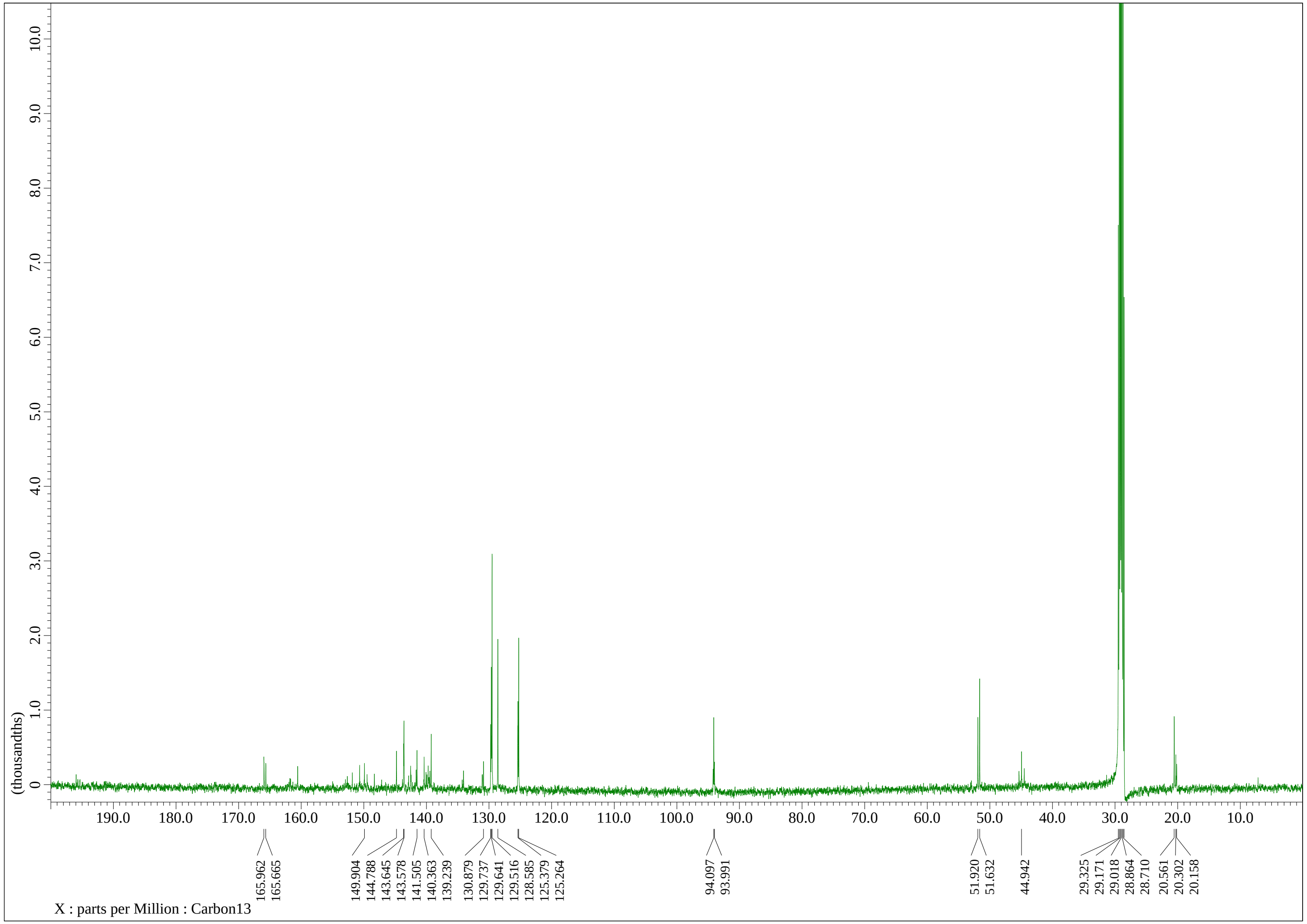

Figure S5a. ^{1}H NMR of 4-PhB(OH)$_2$ complex (acetone-d6)

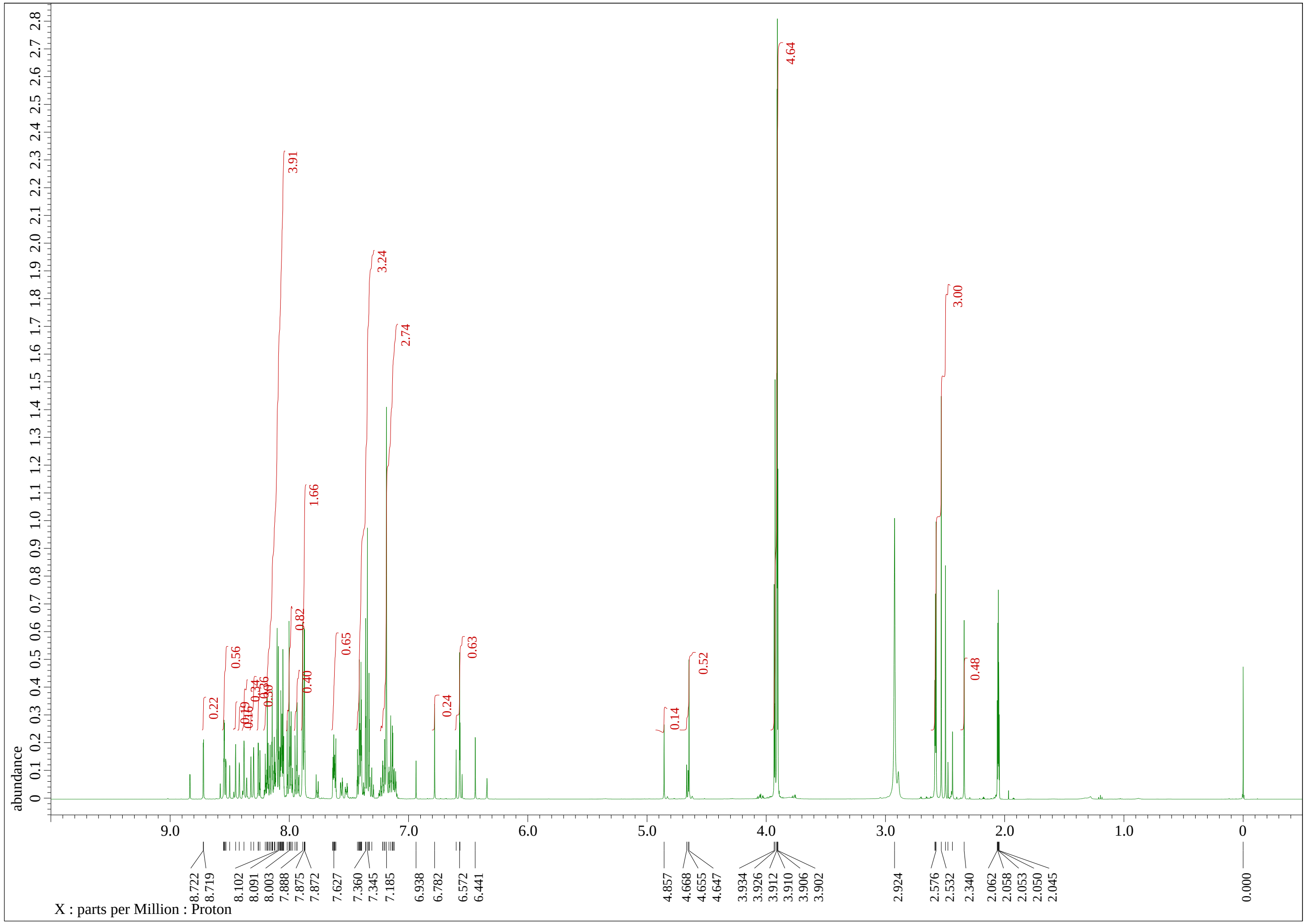

Figure S5b. ^{1}H NMR of 5-PhB(OH)$_2$ complex (acetone-d6, expansion (6.0-9.5ppm))

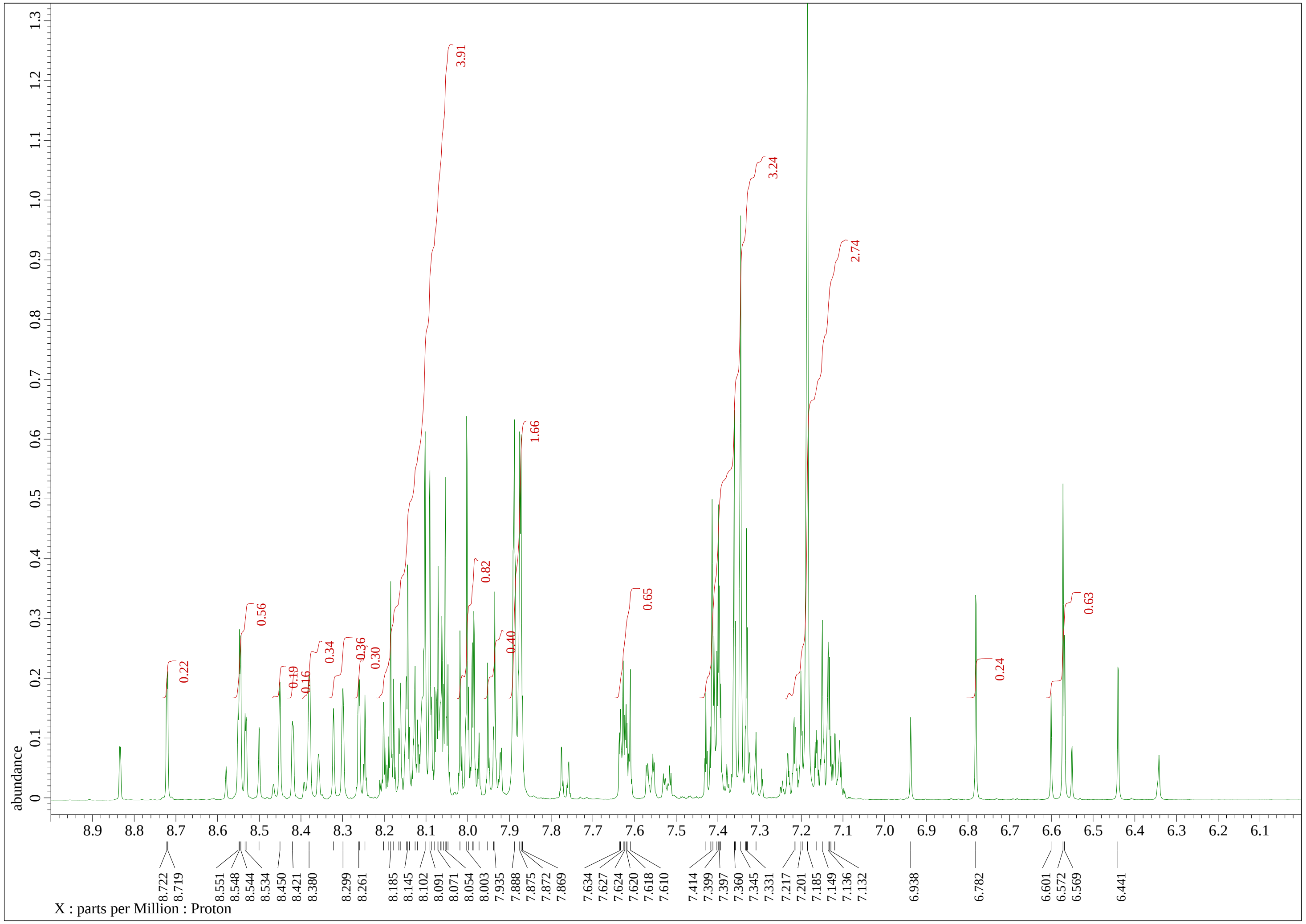

Figure S5c. ^{13}C NMR of 4-PhB(OH)$_2$ complex (acetone-d6)

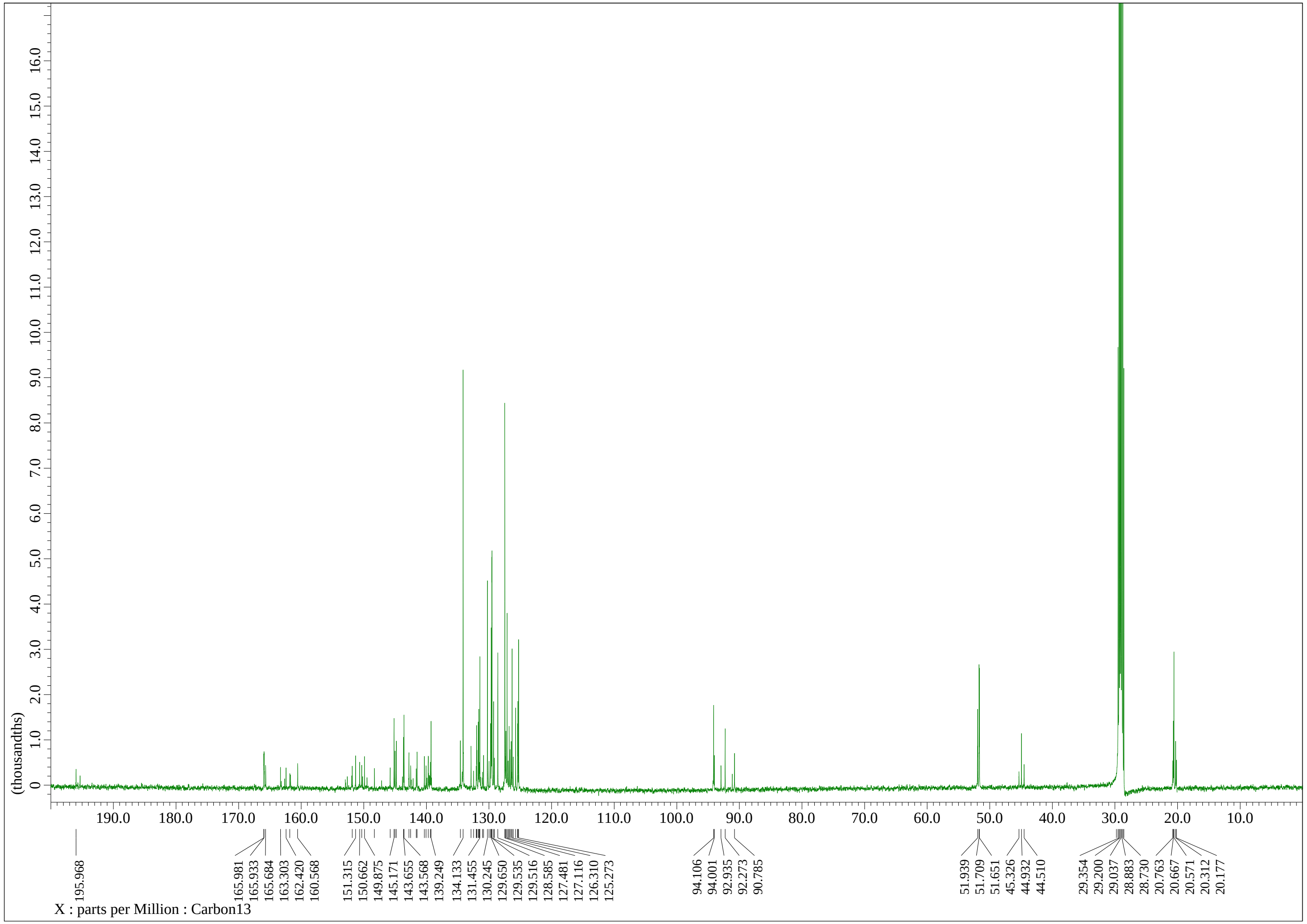

Figure S6a. ^{1}H NMR of 5 (methanol-d4)

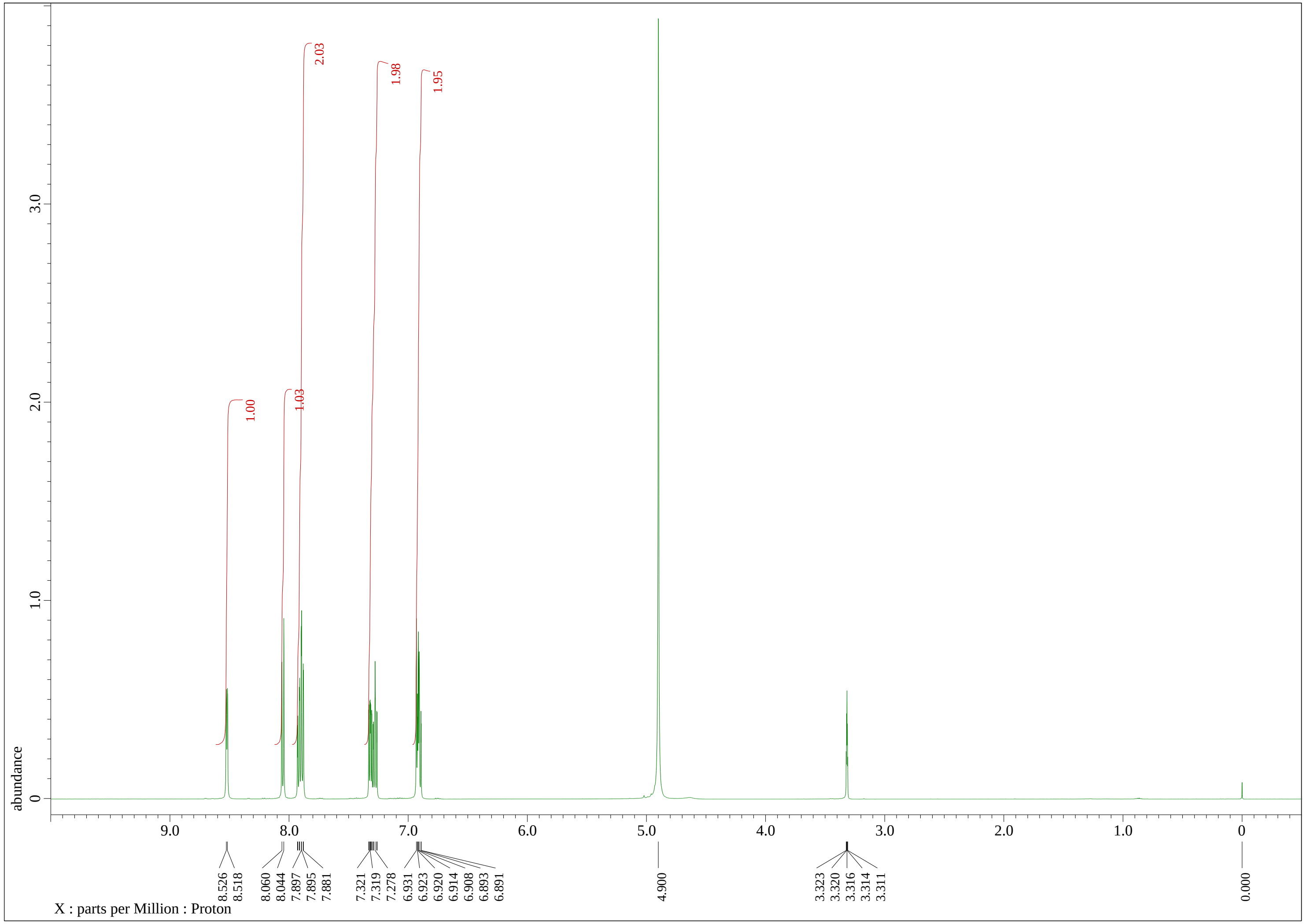

Figure S6b. ^{1}H NMR of 5 (methanol-d4, expansion (6.0-9.0 ppm))

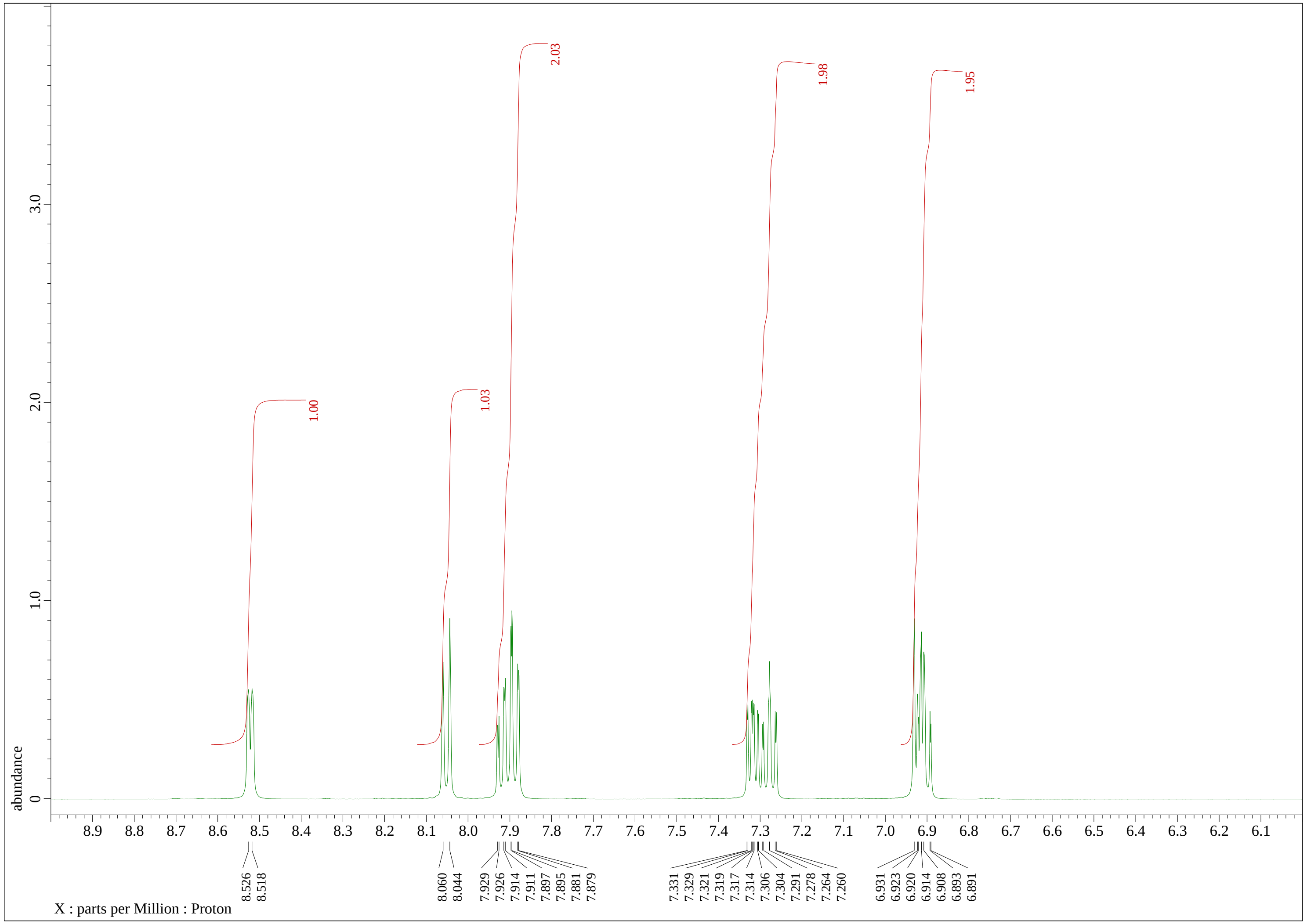

Figure S6c. ^{13}C NMR of 5 (methanol-d4)

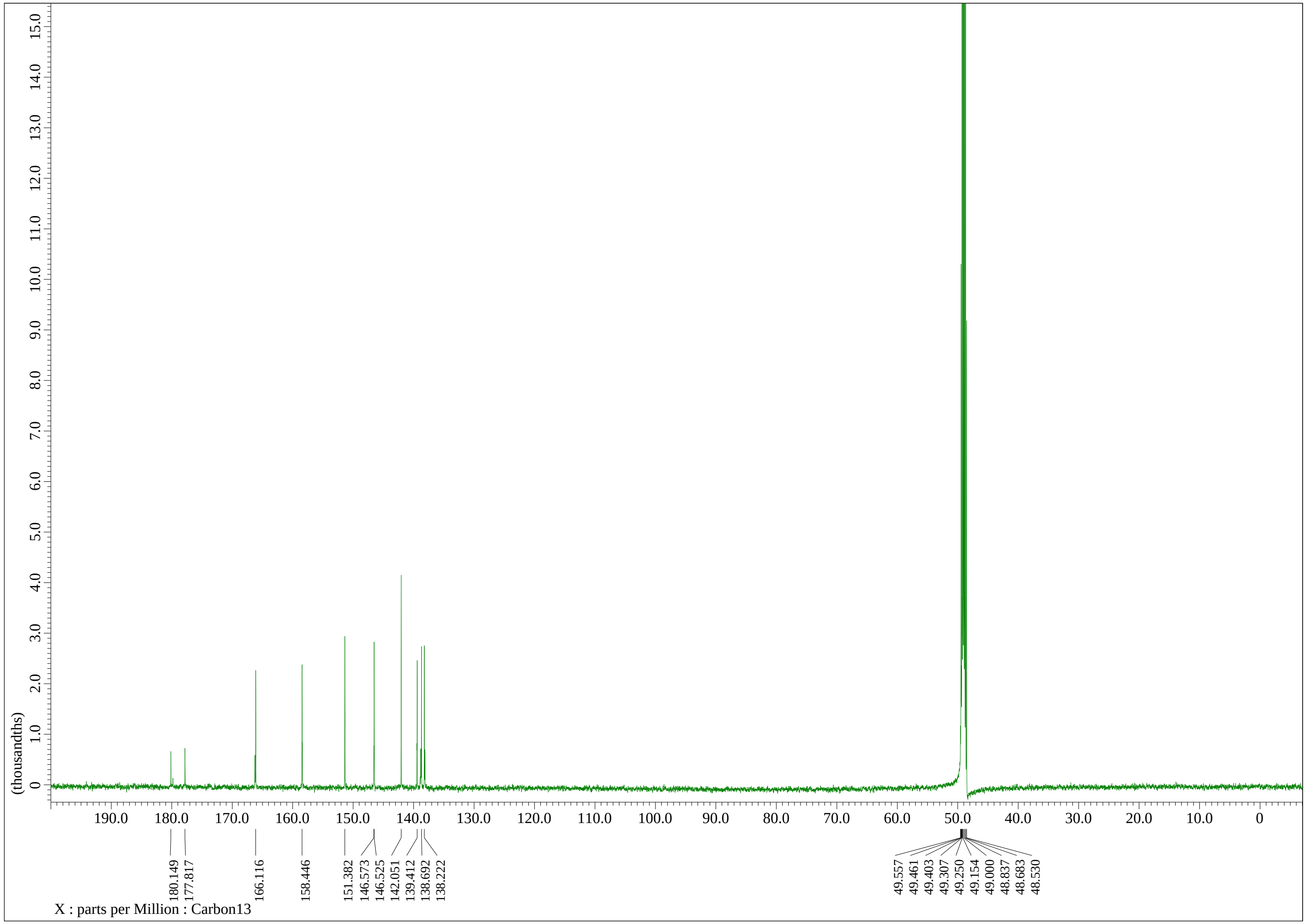

Figure S7a. ^{1}H NMR of 5-PhB(OH)$_2$ complex (methanol-d4)

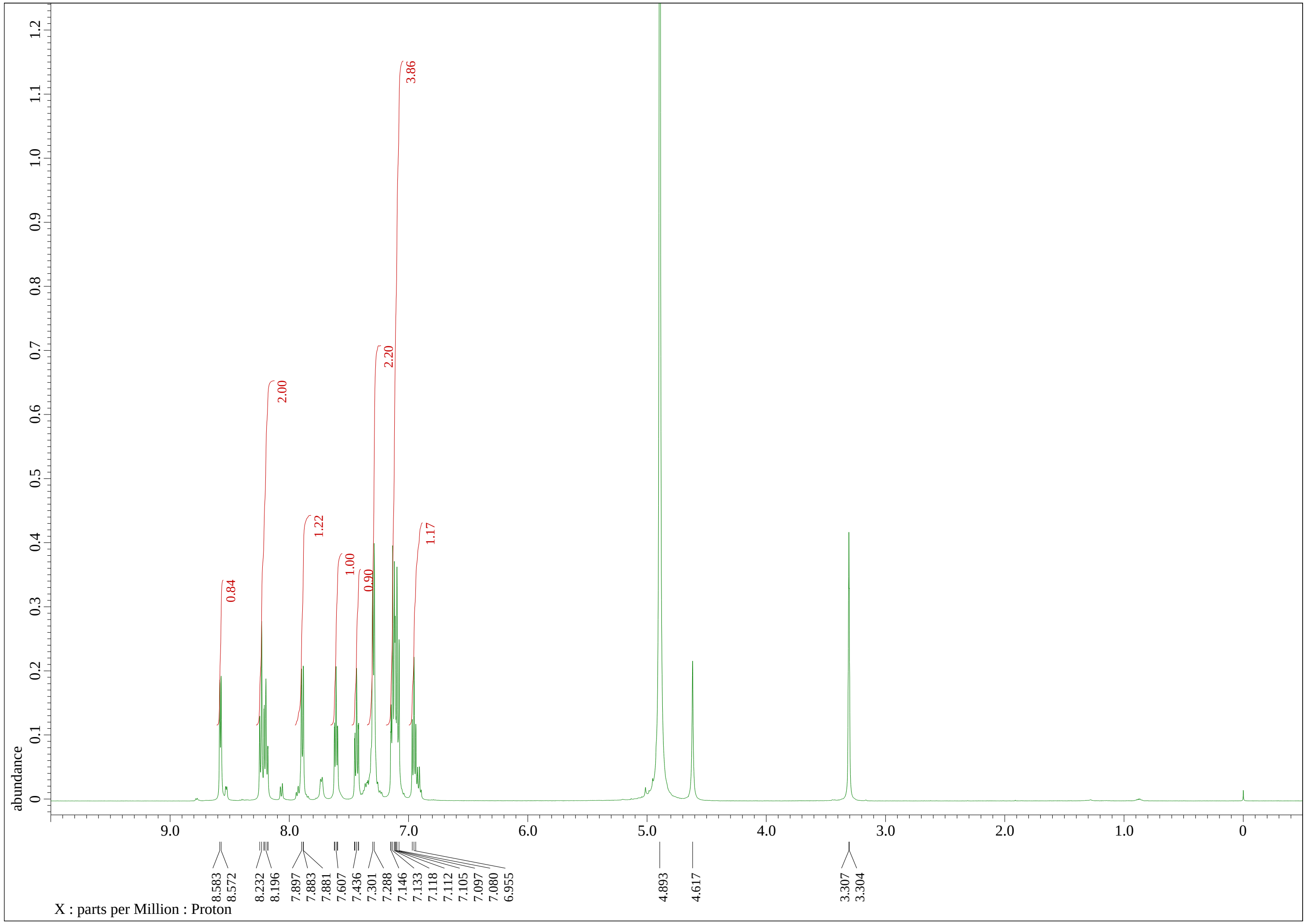

Figure S7b. ^{1}H NMR of 5-PhB(OH)$_2$ complex (methanol-d4, expansion (6.0-9.0ppm))

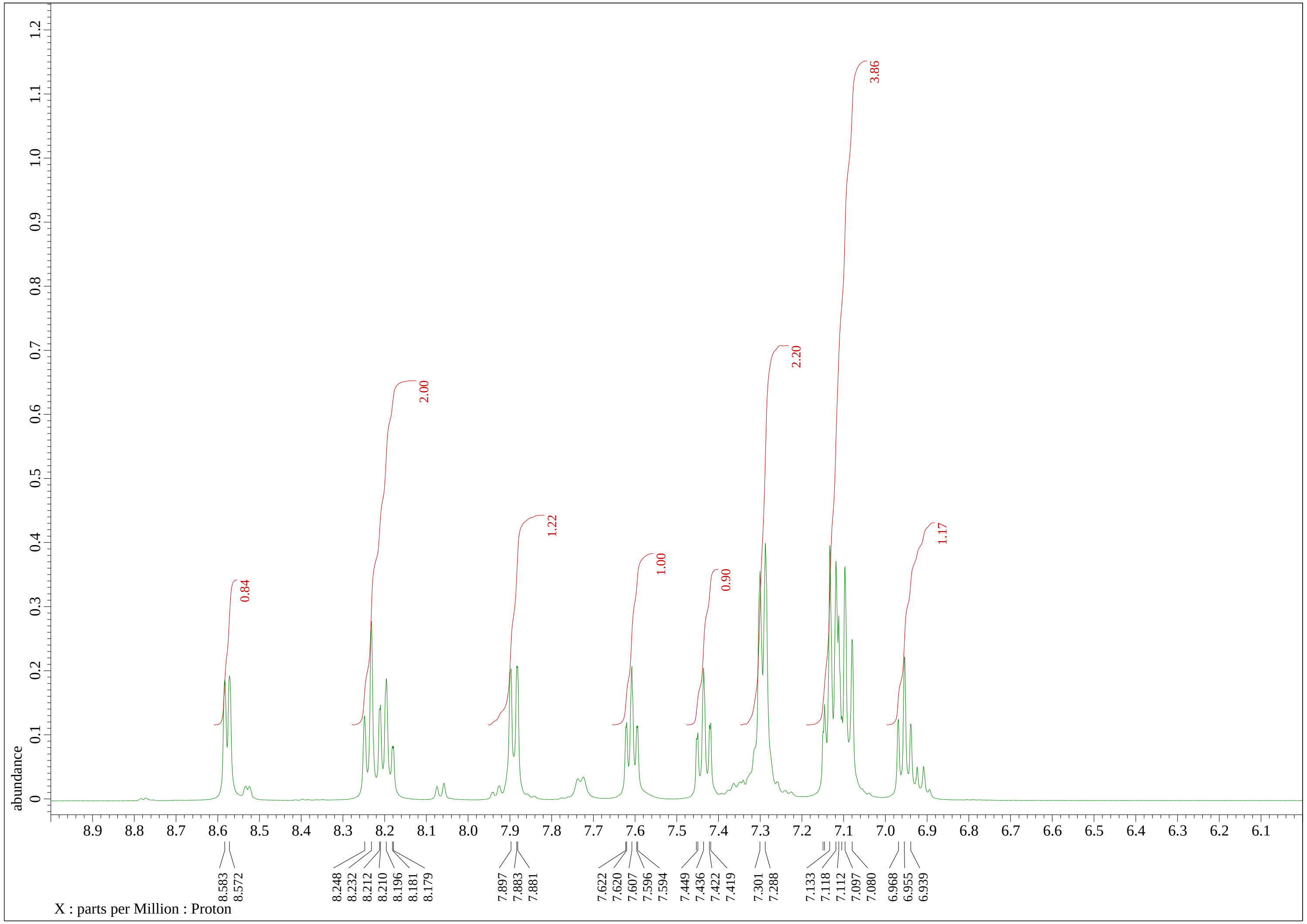

Figure S3. ^{13}C NMR of 5-PhB(OH)$_2$ complex (methanol-d4)

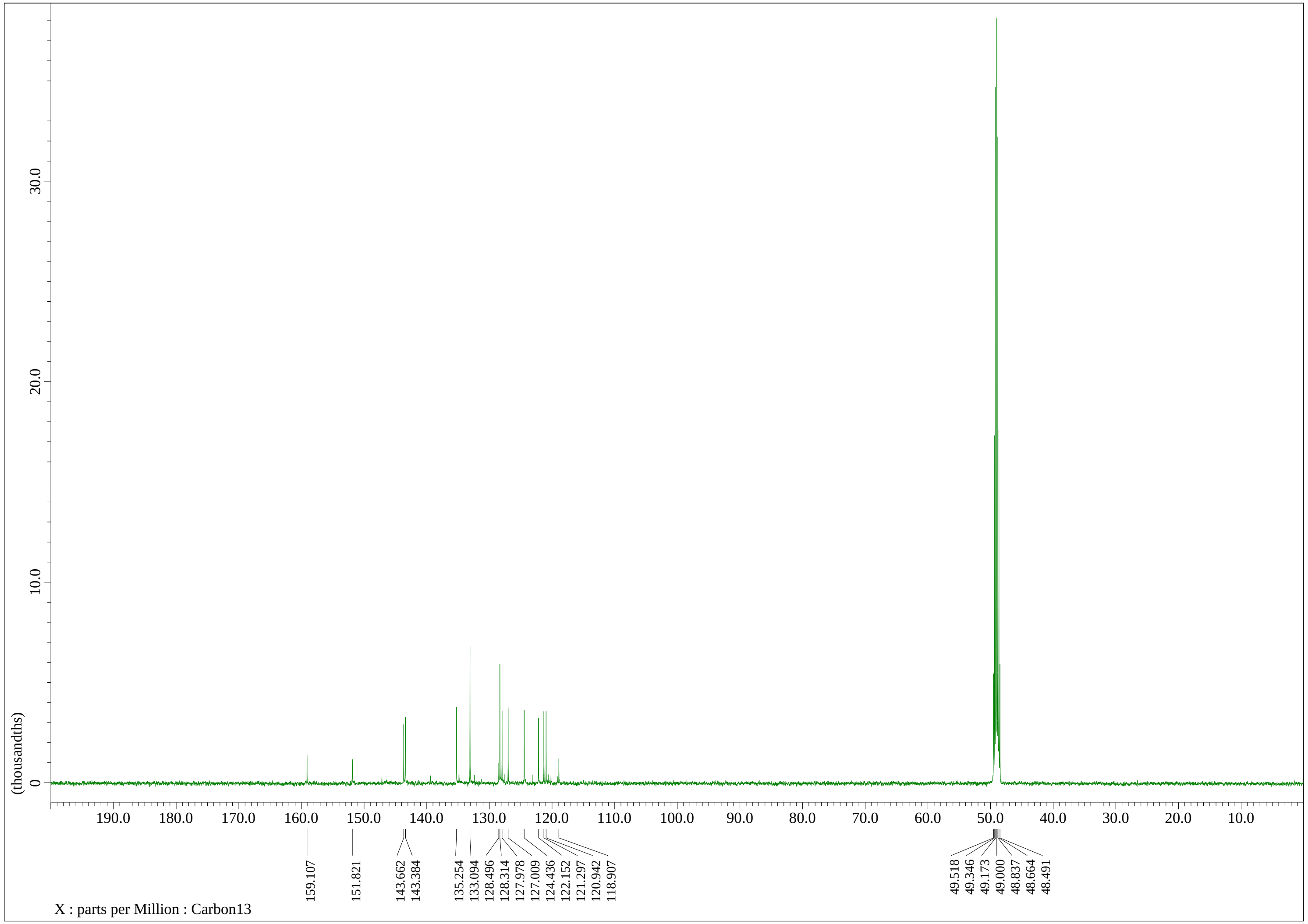

Supplement: Supplementary file 1 [file sensors-17-02436-s001.pdf]
